# CD19 (+) B Cell Combined with Prognostic Nutritional Index Predicts the Clinical Outcomes of Patients with Gastric Cancer Who Underwent Surgery

**DOI:** 10.3390/cancers15092531

**Published:** 2023-04-28

**Authors:** Hao Sun, Huibo Wang, Hongming Pan, Yanjiao Zuo, Ruihu Zhao, Rong Huang, Yingwei Xue, Hongjiang Song

**Affiliations:** Harbin Medical University Cancer Hospital, Harbin Medical University, 150 Haping Road, Nangang District, Harbin 150081, China; haosun@hrbmu.edu.cn (H.S.); 2021021826@hrbmu.edu.cn (H.W.);

**Keywords:** gastric cancer, surgery, peripheral lymphocyte subsets, prognostic nutritional index, prognostic factor

## Abstract

**Simple Summary:**

Gastric cancer has a high degree of malignancy, and even with comprehensive surgical treatment, there is still a high probability of recurrence and metastasis. Finding accurate predictive biomarkers can screen high-risk patients and intervene in a timely manner, which is extremely important for prolonging patient survival. In addition, the value of lymphocyte subset detection in patients with gastric cancer who underwent surgery still needs further exploration. This study further explored the predictive ability of lymphocyte subsets on the prognosis of gastric cancer patients who underwent surgery on a larger sample size and explored the prognostic value of CD19 (+) B cell combined with the Prognostic Nutritional Index (PNI). The results showed that lymphocyte subsets were related to the clinical outcome, the combined index had a stronger prognostic predictive ability than single markers and other non-invasive biomarkers, and was a powerful predictive biomarker for gastric cancer patients who underwent surgery.

**Abstract:**

(1) Background: The aim of this study was to explore the predictive ability of lymphocyte subsets for the prognosis of gastric cancer patients who underwent surgery and the prognostic value of CD19 (+) B cell combined with the Prognostic Nutritional Index (PNI). (2) Methods: This study involved 291 patients with gastric cancer who underwent surgery at our institution between January 2016 and December 2017. All patients had complete clinical data and peripheral lymphocyte subsets. Differences in clinical and pathological characteristics were examined using the Chi-square test or independent sample *t*-tests. The difference in survival was evaluated using Kaplan–Meier survival curves and the Log-rank test. Cox’s regression analysis was performed to identify independent prognostic indicators, and nomograms were used to predict survival probabilities. (3) Results: Patients were categorized into three groups based on their CD19 (+) B cell and PNI levels, with 56 cases in group one, 190 cases in group two, and 45 cases in group three. Patients in group one had a shorter progression-free survival (PFS) (HR = 0.444, *p* < 0.001) and overall survival (OS) (HR = 0.435, *p* < 0.001). CD19 (+) B cell–PNI had the highest area under the curve (AUC) compared with other indicators, and it was also identified as an independent prognostic factor. Moreover, CD3 (+) T cell, CD3 (+) CD8 (+) T cell, and CD3 (+) CD16 (+) CD56 (+) NK T cell were all negatively correlated with the prognosis, while CD19 (+) B cell was positively associated with the prognosis. The C-index and 95% confidence interval (CI) of nomograms for PFS and OS were 0.772 (0.752–0.833) and 0.773 (0.752–0.835), respectively. (4) Conclusions: Lymphocyte subsets including CD3 (+) T cell, CD3 (+) CD8 (+) T cell, CD3 (+) CD16 (+) CD56 (+) NK T cell, and CD19 (+) B cell were related to the clinical outcomes of patients with gastric cancer who underwent surgery. Additionally, PNI combined with CD19 (+) B cell had higher prognostic value and could be used to identify patients with a high risk of metastasis and recurrence after surgery.

## 1. Introduction

According to statistical data, gastric cancer continued to be the fifth most common type of cancer globally and was the third leading cause of cancer-related deaths, surpassed only by lung and liver cancers [1,2]. Currently, surgery is the primary treatment for gastric cancer. However, the recurrence and mortality rates for patients with gastric cancer remains high even after radical resection [3,4]. Thus, it is crucial to investigate effective non-invasive prognostic indicators.

The immune system is essential in preventing and resisting the occurrence and progression of tumors [5]. Normally, it can detect and eliminate abnormal cells in the body, including cancer cells [6]. When the immune system identifies abnormal cells, it triggers a complex cascade of cellular and molecular signals that activate immune cells to initiate an immune response and ultimately eliminate these abnormal cells [7,8,9]. Therefore, patients with a weakened immune status are more likely to experience tumor recurrence [10]. Detection techniques for lymphocyte subsets emerged many years ago. However, their limited reference values for surgery and high price have hindered their usage in gastric cancer patients receiving surgery. Unlike tumor-infiltrating lymphocytes, peripheral lymphocyte subsets are more easily detectable and can also serve as a reflection of a patient’s immune function [11,12]. Previous studies have demonstrated that lymphocyte subsets are reliable biomarkers for cancer patients and are significantly associated with treatment outcomes and prognosis, but these studies were based on a small sample size, and the results need to be further validated [11,13,14,15]. The relationship between nutritional status and tumors is closely intertwined, with many cancer patients experiencing malnutrition due to metabolic changes, anorexia, nausea, vomiting, and other factors resulting from tumor growth and treatment. This is especially true for patients with gastric cancer [16,17,18]. Malnutrition can adversely affect the efficacy of tumor treatment and diminish the body’s immune function, which, in turn, lowers its resistance to tumors and accelerates their growth [19,20]. The immune function of patients is closely linked to their nutritional status, meaning that proper nutrition is essential for maintaining optimal immune function.

The Prognostic Nutritional Index (PNI) can effectively indicate the nutritional and inflammatory status of patients, with numerous studies confirming its effectiveness in assessing gastric cancer [21,22]. By combining PNI, which indicates nutritional and inflammatory status, with lymphocyte subsets that reflect immune status, a more comprehensive evaluation of the condition of gastric cancer patients can be achieved.

## 2. Materials and Methods

### 2.1. Patients

We continuously collected data from 291 patients with gastric cancer who underwent surgery at our institution between January 2016 and December 2017. All patients underwent peripheral lymphocyte subset proportion testing and had complete clinical data. Clinical and pathological information were gathered using an electronic medical records system, and due to the retrospective nature of the study, the Ethics Committee of Harbin Medical University Cancer Hospital waived the need for informed consent (Ethics number: 2019-57-IIT). All analyses were conducted in accordance with the Helsinki Declaration and its amendments.

### 2.2. Data Collection

The study’s endpoints were progression-free survival (PFS) and overall survival (OS), which were determined through centralized telephone follow-up conducted in December 2021. PFS was the period between the beginning of surgery and the progression of the disease, and evidence of disease progression was determined through imaging tests such as enhanced CT. For patients without evidence of disease progression, PFS also ended at the time of the last follow-up. OS was the period from the beginning of surgery to death or the last follow-up.

### 2.3. Peripheral Lymphocyte Subsets and PNI

**Figure 1:**
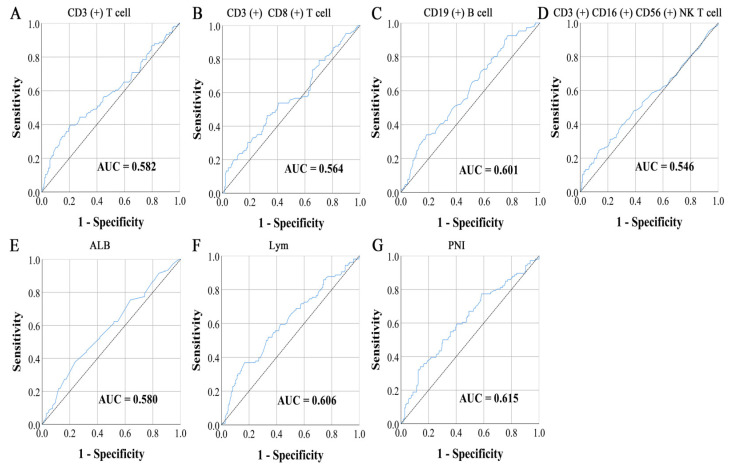
The ROC curve of (**A**) CD3 (+) T cell, (**B**) CD3 (+) CD8 (+) T cell, (**C**) CD19 (+) B cell, (**D**) CD3 (+) CD16 (+) CD56 (+) NK T cell, (**E**) ALB, (**F**) Lym, and (**G**) PNI.

The percentage of peripheral lymphocyte subsets were detected via flow cytometry and including CD3 (+) T cell, CD3 (+) CD4 (+) T cell, CD3 (+) CD8 (+) T cell, CD3 (+) CD4 (+) CD8 (+) T cell, CD19 (+) B cell, CD3 (−) CD16 (+) CD56 (+) NK cell, and CD3 (+) CD16 (+) CD56 (+) NK T cell. In addition, we also calculated the ratio of CD4 to CD8. The sum of their proportions was approximately equal to 100%. PNI was calculated as follows: PNI = albumin (g/L) + 5 × lymphocyte (10^9^/L). The cut-off points for CD19 (+) B cell and PNI were obtained using the maximum Youden index [Sensitivity − (1 − Specificity)] calculated by the receiver operating characteristic (ROC) curve. The maximum Youden indexes for CD19 (+) B cell and PNI were 0.157 and 0.199, and their cut-off values were 15.40% and 45.82 (Figure 1C,G). Patients with CD19 (+) B cell levels < 15.40% and PNI < 45.82 were included in group 1, those with CD19 (+) B cell levels ≥ 15.40% and PNI ≥ 45.82 were placed in group 3, while the remaining cases were categorized under group 2.

### 2.4. Statistical Analysis

We performed all statistical analyses using R version 4.2.2 (https://www.r-project.org, accessed on 2 March 2023) and GraphPad Prism 8.0 (https://www.graphpad.com, accessed on 3 March 2023). Statistical significance was set at a two-sided *p* value of <0.05. Differences in clinical information were compared using Student’s *t*-test, Chi-square test, or Fisher’s exact test. Survival differences were evaluated using Kaplan–Meier survival curves and Log-rank test. Cox’s regression analysis was conducted to identify prognostic markers, with relative risks estimated by the hazard ratio (HR) and 95% confidence interval (CI). Finally, we developed nomograms to predict the survival probability of patients and assessed their predictive performance using calibration curves.

## 3. Results

### 3.1. Patient Characteristics

This study enrolled a total of 291 cases, with 203 (69.8%) men and 88 (30.2%) women, and a mean age of 59.05 (10.45) years. All patients underwent surgery, with 274 patients (94.2%) receiving radical resection. Due to non-normal distribution of tumor markers, patients were categorized into two groups based on the median of tumor markers. Our results showed that CD19 (+) B cell–PNI was associated with age, body mass index (BMI), TNM stage, and CA724 (all *p* < 0.05) (Table 1).

Furthermore, Fisher’s exact test revealed that patients in group one tended to have larger tumor sizes (*p* < 0.001). When analyzing blood parameters, we found that cases with low CD19 (+) B cell and PNI had lower γ-glutamyl transferase (γ-GGT), lower total bilirubin (TBIL), lower indirect bilirubin (IDBIL), lower total protein (TP), lower albumin (ALB), lower globulin (GLOB), lower prealbumin (PALB), lower lymphocyte (Lym), higher CD3 (+) T cell, higher CD3 (+) CD8 (+) T cell, lower CD19 (+) B cell, and higher CD3 (−) CD16 (+) CD56 (+) NK cell (all *p* < 0.05) (Table 2).

### 3.2. Univariate and Multivariate Cox’s Regression Analysis

We conducted Cox’s regression analysis on the clinical and pathological information of patients. In addition, to explore the impact of lymphocyte subsets more accurately on prognosis, we have also included non-grouped lymphocyte subsets in the analysis. The results showed that age, BMI, CD3 (+) CD8 (+) T cell, CD19 (+) B cell, CD3 (+) CD16 (+) CD56 (+) NK T cell, ALB, Lym, PNI, CD19 (+)-B cell–PNI, radical resection, Borrmann type, lymph node positive (LNP), tumor size, and TNM stage (all *p* < 0.05) were significantly associated with both PFS and OS. Furthermore, CD3 (+) T cell was also identified as a prognostic factor for OS (*p* = 0.028). After incorporating meaningful indicators from univariate analysis into Cox’s multivariate regression analysis, we found that age, CD19 (+) B cell–PNI, and TNM stage were identified as independent prognostic markers for both PFS and OS (all *p* < 0.05) (Table 3 and Table 4).

In addition, we evaluated the predictive advantage of different parameters for prognosis using AUC calculated by ROC with death as the endpoint. At the same time, to highlight the prognostic value of combined indicators, we also included classic inflammation and nutritional markers in the analysis. Their calculation formulas are shown in Table 5. The results showed that CD19 (+) B cell had the highest area under curve (AUC) in lymphocyte subsets and PNI had the highest AUC in classic inflammatory and nutritional markers. The combined indicators, consisting of CD19 (+) B cell and PNI, demonstrated a significant advantage in predicting prognosis among non-invasive biomarkers (AUC = 0.648) (Table 6).

### 3.3. Survival Analysis for Lymphocyte Subsets

**Figure 2:**
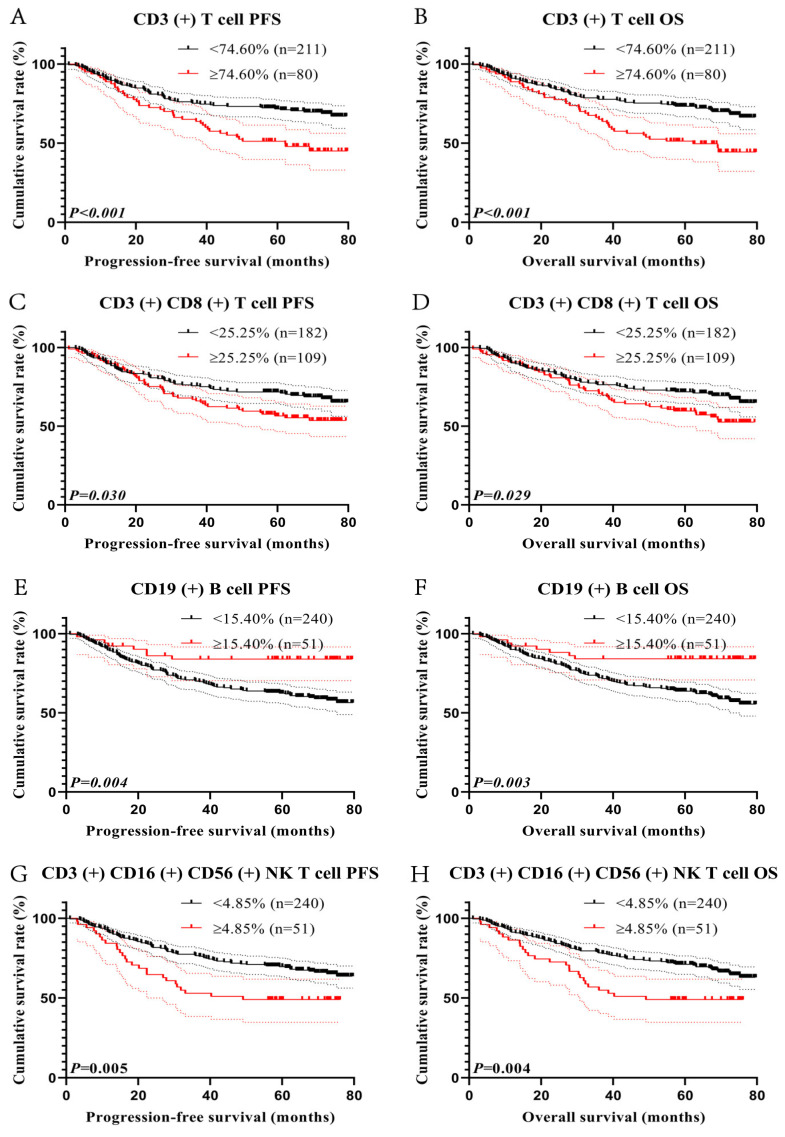
Survival curve for lymphocyte subset. CD3 (+)-related survival curve for (**A**) PFS and (**B**) OS; CD3 (+) CD8 (+)-related survival curve for (**C**) PFS and (**D**) OS; CD19 (+)-related survival curve for (**E**) PFS and (**F**) OS. CD3 (+) CD16 (+) CD56 (+)-related survival curve for (**G**) PFS and (**H**) OS.

As some of the lymphocyte subset indicators were found to be related to survival in Cox’s regression analysis, the maximum Youden indexes for CD3 (+) T cell, CD3 (+) CD8 (+) T cell, and CD3 (+) CD16 (+) CD56 (+) NK T cell were 0.191, 0.138, and 0.110, and their cut-off values were 74.60%, 25.25%, and 4.85% (Figure 1A,B,D). There were 211 patients with CD3 (+) T cell < 74.60%, with 1-, 3-, and 5-year survival rates for PFS and OS of 90.5%, 75.2%, and 71.7%, and 91.0%, 77.7%, and 73.3%, respectively. There were 80 patients with CD3 (+) T cell ≥ 74.60%, with 1-, 3-, and 5-year survival rates for PFS and OS of 88.8%, 65.0%, and 51.2%, and 89.7%, 78.6%, and 75.2%, respectively. Patients with high CD3 (+) T cell levels had a shorter PFS (HR = 1.995, *p* < 0.001) and OS (HR = 2.051, *p* < 0.001) (Figure 2A,B).

After grouping, 182 cases were enrolled in the CD3 (+) CD8 (+) T cell < 25.25% group and 109 cases were enrolled in the CD3 (+) CD8 (+) T cell ≥ 25.25% group. The 1-, 3-, and 5-year survival rates for PFS in patients with CD3 (+) CD8 (+) T cell < 25.25% and CD3 (+) CD8 (+) T cell ≥ 25.25% were 90.8%, 75.6%, and 71.7% and 89.6%, 67.0%, and 56.5%, respectively. The corresponding survival rates for OS were 91.8%, 76.3%, and 71.8% and 90.1%, 71.6%, and 59.6%. Notably, patients with high CD3 (+) CD8 (+) T cell were associated with poorer PFS (HR = 1.513, *p* = 0.030) and OS (HR = 1.516, *p* = 0.029) (Figure 2C,D).

There were 240 patients with CD19 (+) B cell < 15.40%, and their 1- and 3-year survival rates for PFS and OS were 89.6% and 90.0%, respectively, while there were 51 patients with CD19 (+) B cell ≥ 15.40%, and their 1- and 3-year survival rates for PFS and OS were 92.2% and 83.9% and 92.2% and 84.1%, respectively. Patients with low CD19 (+) B cell had shorter PFS (HR = 0.358, *p* < 0.004) and OS (HR = 0.351, *p* < 0.003) (Figure 2E,F).

There were then 240 cases with CD3 (+) CD16 (+) CD56 (+) NK T cell < 4.85% and 51 cases with CD3 (+) CD16 (+) CD56 (+) NK T cell ≥ 4.85%. Patients with CD3 (+) CD16 (+) CD56 (+) NK T cell < 4.85% had 1-, 3-, and 5-year survival rates for PFS and OS of 91.3%, 76.5%, and 69.6% and 91.3%, 78.3%, and 71.1%, respectively. In addition, patients with CD3 (+) CD16 (+) CD56 (+) NK T cell ≥ 4.85% had 1-, 3-, and 5-year survival rates for PFS and OS of 84.3%, 52.9%, and 49.0% and 86.3%, 56.9%, and 48.5%, respectively. Patients with high CD3 (+) CD16 (+) CD56 (+) NK T cell had significantly poorer PFS (HR = 1.865, *p* = 0.005) and OS (HR = 1.880, *p* = 0.004) (Figure 2G,H).

### 3.4. Survival Analysis for Prognostic Nutritional Index

**Figure 3:**
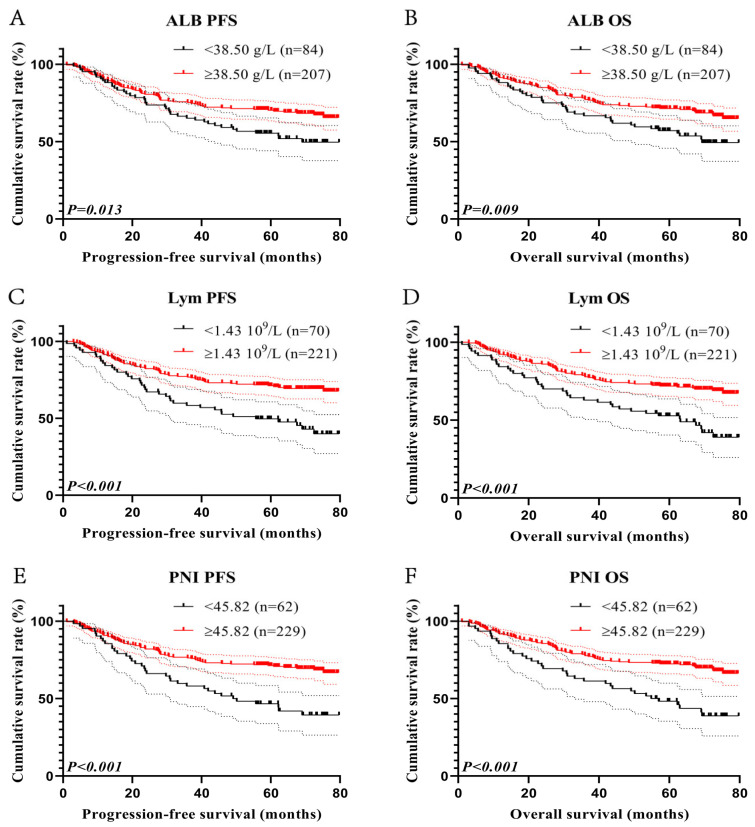
Survival curve for Prognostic Nutritional Index. ALB-related survival curve for (**A**) PFS and (**B**) OS; Lym-related survival curve for (**C**) PFS and (**D**) OS; PNI-related survival curve for (**E**) PFS and (**F**) OS.

In this study, we conducted a survival analysis for PNI because it has the highest ACU among classic inflammatory and nutritional markers. The maximum Youden indexes calculated by ROC for ALB and Lym were 0.140 and 0.200, and their cut-off values were 38.50 g/L and 1.43 × 10^9^/L (Figure 1E,F). Of the total 291 patients, there were 84 cases with ALB < 38.50 g/L and 207 cases with ALB ≥ 38.50 g/L. The 1-, 3-, and 5-year survival rates for both PFS and OS in patients with ALB < 38.50 g/L were 89.3%, 65.2%, and 55.4% vs. 88.1%, 66.7%, and 57.1%, respectively. In addition, the corresponding survival rates in patients with ALB ≥ 38.50 g/L were 90.3%, 75.3%, and 70.2% vs. 91.3%, 77.7%, and 71.3%. Patients with low ALB levels had significantly shorter PFS and OS (HR = 0.600, *p* = 0.013 and HR = 0.583, *p* = 0.009, respectively) (Figure 3A,B).

There were 70 patients with Lym < 1.43 10^9^/L and 221 patients with Lym ≥ 1.43 × 10^9^/L. The 1-, 3-, and 5-year survival rates for PFS in patients with Lym < 1.43 × 10^9^/L and Lym ≥ 1.43 × 10^9^/L were 85.7%, 58.4%, and 49.6% and 91.4%, 76.8%, and 71.2%, respectively. Similarly, the corresponding survival rates for OS were 84.3%, 62.9%, and 52.8% and 92.3%, 78.2%, and 71.8%. Patients with low Lym had poorer PFS and OS (HR = 0.456, *p* < 0.001 and HR = 0.453, *p* < 0.001) (Figure 3C,D).

There were 62 patients with PNI < 45.82, with 1-, 3-, and 5-year survival rates for PFS and OS of 87.1%, 58.1%, and 46.6% and 85.5%, 61.3%, and 48.3%. Meanwhile, there were 229 patients with PNI ≥ 45.82, with 1-, 3-, and 5-year survival rates for PFS and OS of 90.8%, 76.3%, and 71.3% and 91.7%, 78.1%, and 72.3%. Patients with PNI < 45.82 also related to shorter PFS (HR = 0.441, *p* < 0.001) and OS (HR = 0.431, *p* < 0.001) (Figure 3E,F).

### 3.5. Survival Analysis for CD19 (+) B Cell–PNI

**Figure 4:**
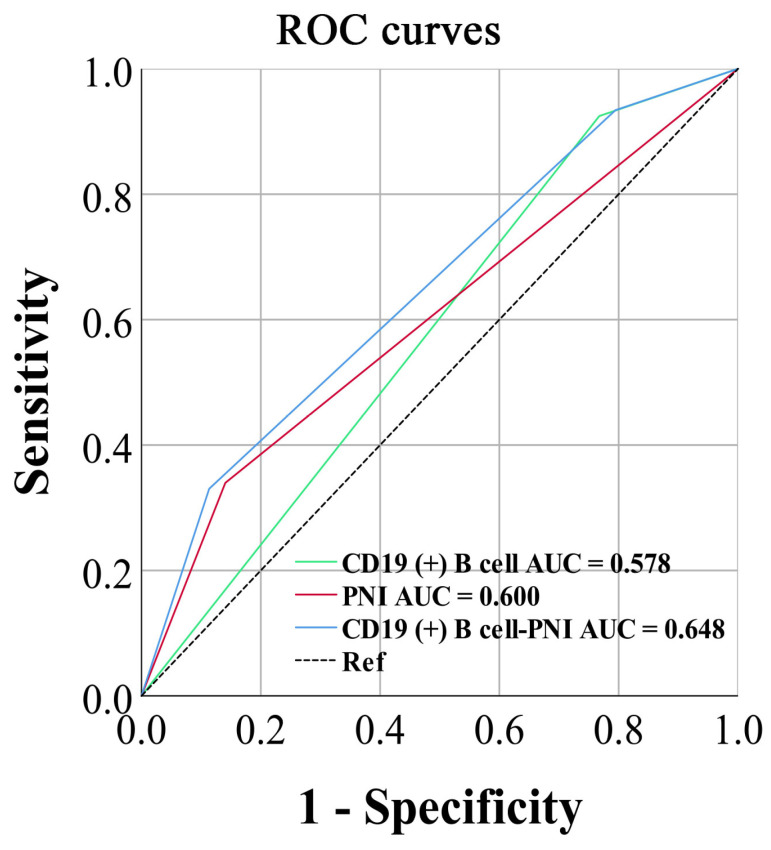
The ROC curve and AUC of grouped CD19 (+) B cell–PNI-related factors.

Due to the higher AUC of CD19 (+) B cell and PNI, we analyzed their relevant indicators and combined them for survival analysis. We also compared the ROC curves of grouped CD19 (+) B cell, PNI, and CD19 (+) B cell–PNI, and found that the AUC for PNI was 0.615, that for CD19 (+) B cell was 0.601, and that for CD19 (+) B cell–PNI was 0.648. The CD19 (+) B cell–PNI also had a higher AUC, indicating that it had a higher prognostic value compared with a single indicator (Figure 4).

**Figure 5:**
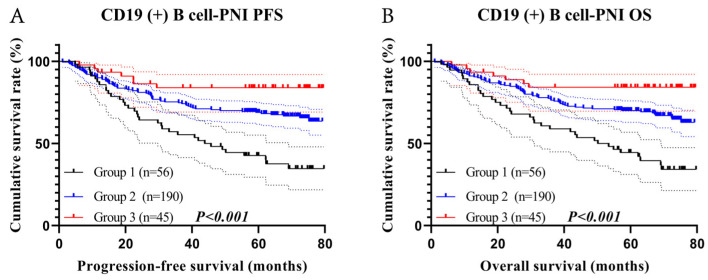
CD19 (+) B cell–PNI-related survival curve of (**A**) PFS and (**B**) OS.

We grouped patients as follows: 56 cases in group one with 1-, 3-, and 5-year survival rates of 87.5%, 55.4%, and 42.7% for PFS and 85.7%, 58.9%, and 44.5% for OS; 190 cases in group two with 1-, 3-, and 5-year survival rates of 90.0%, 74.7%, and 68.9% for PFS and 91.1%, 76.8%, and 70.0% for OS; and 45 cases in group three with 1-, 3-, and 5-year survival rates of 93.3%, 83.9%, and 73.1% for PFS and 93.1%, 84.2%, and 73.3% for OS. Patients in group one had shorter PFS (HR = 0.444, *p* < 0.001) and OS (HR = 0.435, *p* < 0.001) (Figure 5A,B).

### 3.6. Survival Analysis for CD19 (+) B Cell–PNI in Different TNM Stages

**Figure 6:**
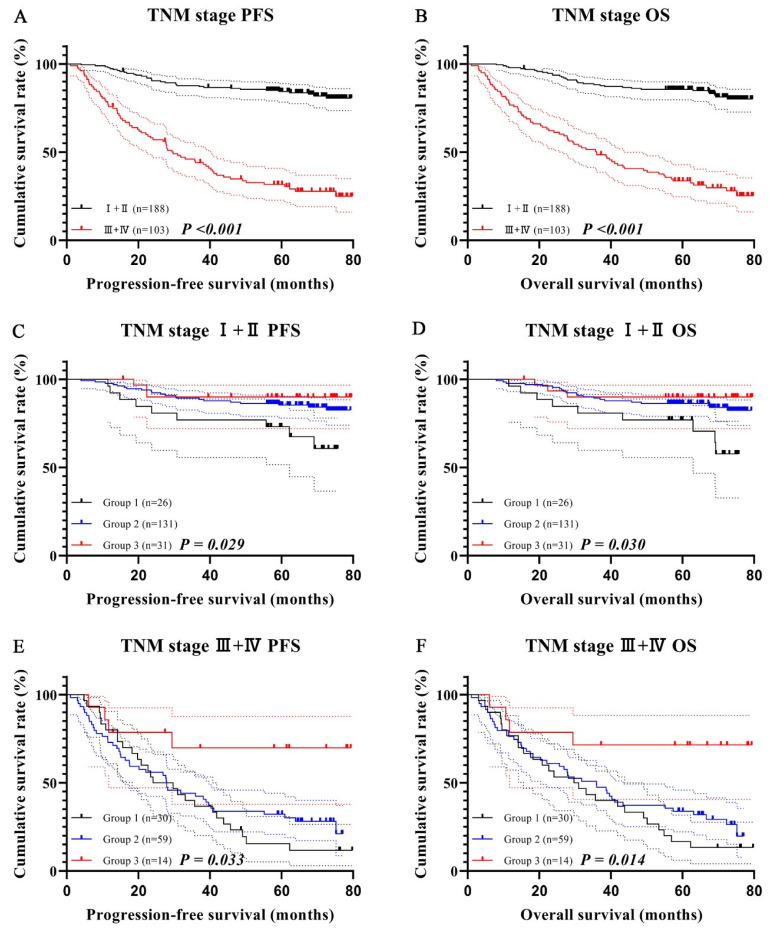
CD19 (+) B cell–PNI-related survival curves in different TNM stages. TNM-stage-related survival curve for PFS (**A**) and OS (**B**); CD19 (+) B cell–PNI-related survival curves in TNM stages I and II for PFS (**C**) and OS (**D**); CD19 (+) B cell–PNI-related survival curves in TNM stages III and IV for PFS (**E**) and OS (**F**).

As the patients in this study were at different TNM stages, we explored the prognostic significance of combined indicators in different TNM stages. Additionally, due to the uneven distribution of CD19 (+) B cell–PNI in different TNM stages, we combined stages I and II, as well as stages III and IV for analysis. There were 188 cases with stage I and II, with 1-, 3-, and 5-year survival rates for PFS and OS of 97.9%, 87.7%, and 84.4% and 97.9%, 88.2%, and 85.6%. Meanwhile, there were 103 patients with stage III and IV, with 1-, 3-, and 5-year survival rates for PFS and OS of 75.7%, 44.0%, and 31.7% and 76.7%, 49.5%, and 33.7%. Patients with stage III and IV closely related to shorter PFS (HR = 6.723, *p* < 0.001) and OS (HR = 6.528, *p* < 0.001) (Figure 6A,B).

In TNM stages I and II, there were 26 cases in group one with 1- and 3-year survival rates of 96.2% and 76.9% for PFS and 96.2% and 80.8% for OS. At the same time, there were 131 cases in group two with 1- and 3-year survival rates of 97.7% and 89.3% for PFS and 97.6% and 88.4% for OS. In addition, there were 31 cases in group three with 1- and 3-year survival rates of 99.9% and 90.0% for PFS and 100.0% and 91.1% for OS. Patients in group one had poorer PFS (HR = 0.466, *p* = 0.029) and OS (HR = 0.468, *p* = 0.030) (Figure 6C,D).

In TNM stages III and IV, there were 30 patients in group one with 1- and 3-year survival rates of 70.1% and 36.7% for PFS and 71.7% and 40.0% for OS. At the same time, there were 59 patients in group two with 1- and 3-year survival rates of 72.9% and 42.4% for PFS and 76.3% and 49.2% for OS. In addition, there were 14 patients in group three with 1- and 3-year survival rates of 78.6% and 69.8% for PFS and 78.6% and 71.4% for OS. Patients in group one also had shorter PFS (HR = 0.611, *p* = 0.033) and OS (HR = 0.570, *p* = 0.014) (Figure 6E,F).

### 3.7. Nomograms

**Figure 7:**
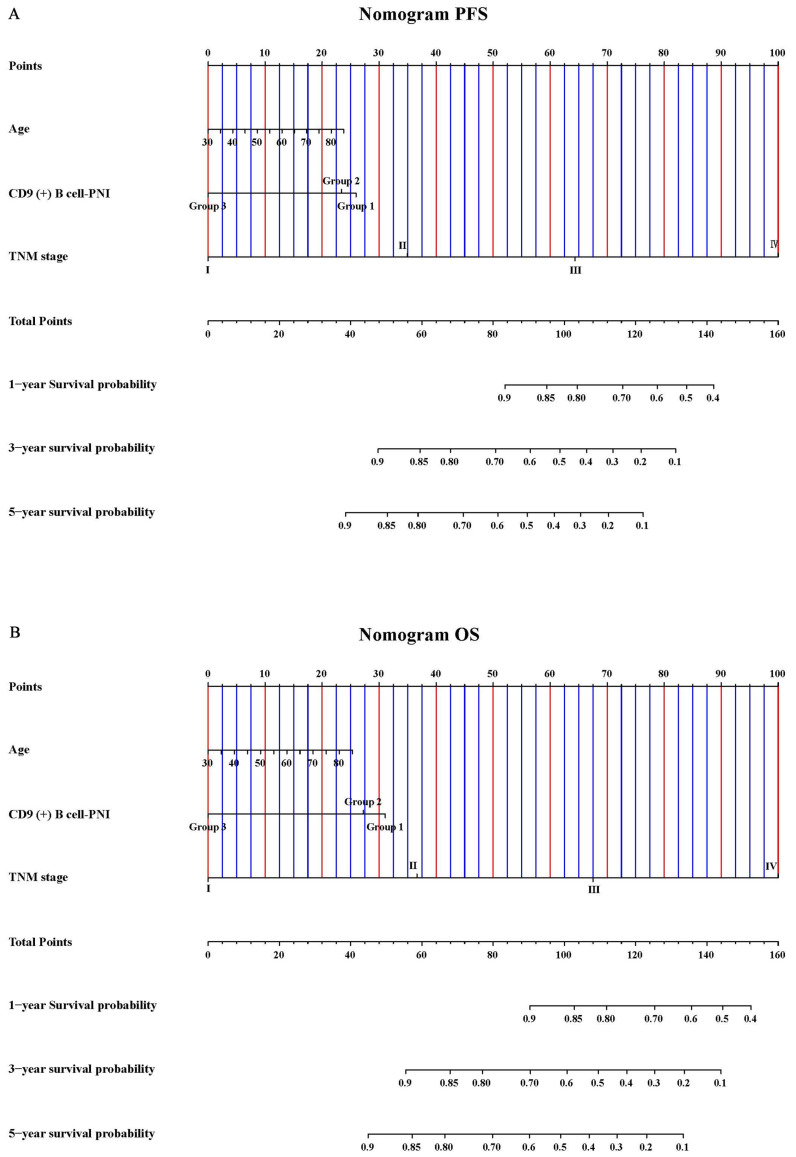
Nomograms of (**A**) PFS and (**B**) OS.

**Figure 8:**
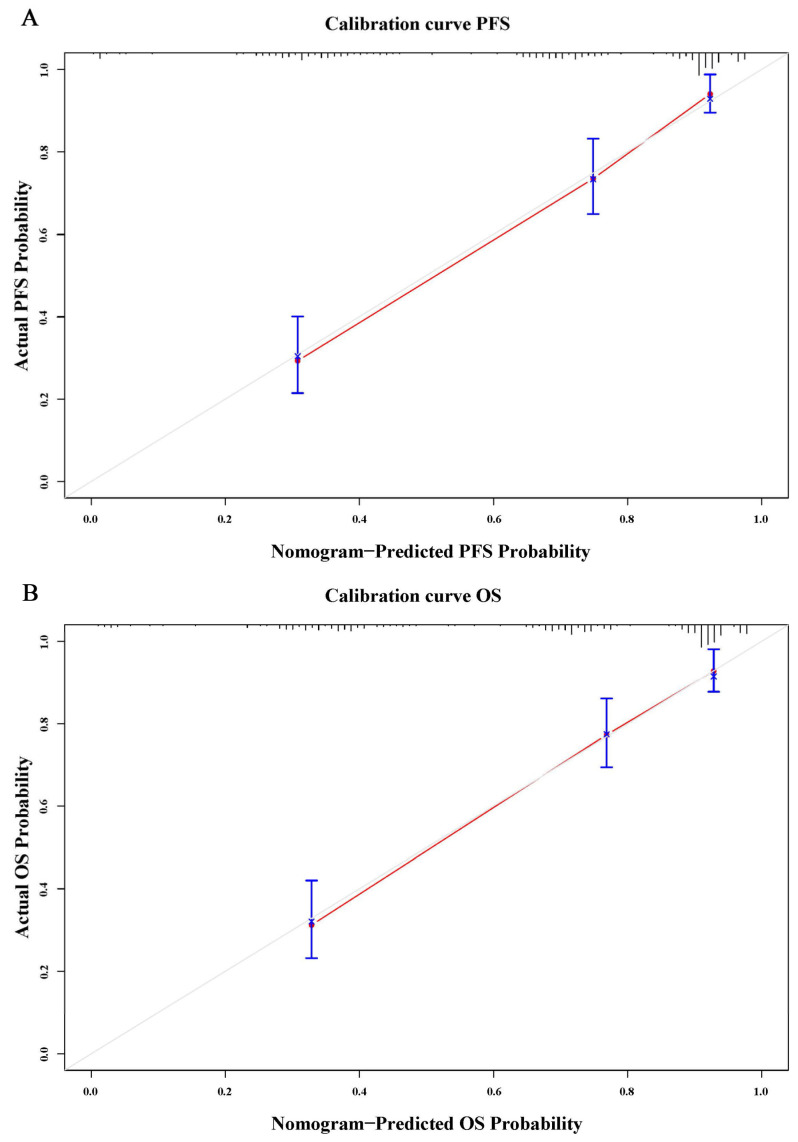
The calibration curves of the nomograms for (**A**) PFS and (**B**) OS.

To further verify the prognostic effectiveness of combined indicators, we constructed nomograms to predict the probability of PFS and OS based on age, CD19 (+) B cell–PNI, and TNM stage (Figure 7A,B). The C-index and 95% CI of the nomograms were 0.772 (0.752–0.833) for PFS and 0.773 (0.752–0.835) for OS. Furthermore, bootstrap correction showed good consistency of the nomograms (Figure 8A,B).

## 4. Discussion

After the discovery that solid tumors can affect the composition and quantity of circulating lymphocyte subpopulations, the relationship between peripheral lymphocyte subpopulations and tumor prognosis has been extensively studied. Zhu and his colleagues gathered data from 220 patients with nasopharyngeal carcinoma who underwent concurrent chemoradiotherapy. They analyzed the patients’ EBV status and peripheral lymphocyte subsets and found that higher levels of CD3 (+) CD8 (+) percentage and lower levels of CD3 (−) CD56 (+) percentage were linked to better OS [23]. In another study, Zhou and his colleagues also discovered the predictive value of certain subpopulations of peripheral lymphocytes. They collected data from 84 patients with stage III esophageal squamous cell carcinoma who had undergone neoadjuvant chemotherapy and analyzed their disease progression. Their analysis revealed that the percentage of NK cells was an independent predictor of pathological complete response [24]. In 2019, Yang and his colleagues studied the predictive ability of circulating lymphocyte subsets for clinical outcomes in metastatic breast cancer. Through survival analysis of 482 patients with metastatic breast cancer, they found that high levels of CD3 (+) T cell and CD3 (+) CD4 (+) T cell were associated with poor outcomes [25]. Peripheral lymphocyte subsets can also predict the prognosis of patients with gastric cancer. Gao et al. collected clinical information and peripheral lymphocyte subset data from 171 patients with gastric cancer who underwent radical resection. Survival analysis revealed that total T-cell count, B-cell count, and percentage of regulatory T-cells were independent predictors of recurrence-free survival [26]. Another study targeting gastric cancer also reached similar conclusions [27]. As a commonly used nutritional biomarker, PNI has been extensively studied and confirmed for its ability to predict the prognosis of gastric cancer [28,29,30,31].

This study further explored the relationship between lymphocyte subsets and prognosis in patients with gastric cancer who underwent surgery on a larger sample size. We performed Cox’s regression analysis on all ungrouped peripheral lymphocyte subset indicators and found that patients with high percentages of CD3 (+) T cells, CD3 (+) CD8 (+) T cells, CD3 (+) CD16 (+) CD56 (+) NK T cells, and low percentages of CD19 (+) B cells had worse PFS and OS. After grouping based on ROC curves, the survival analysis still yielded the same results. This result seems different from previous studies which found that T lymphocyte subsets were positively correlated with the prognosis of cancer patients [23,32]. The possible reason was that the percentage of peripheral lymphocyte subsets could only reflect the changes in the composition of different lymphocyte populations but could not accurately reflect the quantity of a certain lymphocyte. In addition, many gastric cancer patients included in this study were at TNM stages I and II (64.6%). The weak ability of tumor tissue to suppress immune function allows the immune system to maintain a response to the tumor. The main type of tumor immunity is cellular immunity, and an increase in the proportion of T lymphocyte subsets may indicate a high tumor burden in patients [33,34,35].

Due to the close relationship between nutritional status and gastric cancer, we first combined lymphocyte subsets with PNI to determine the status of patients. In extensive analysis of the prognostic value of various parameters, we found that CD19 (+) B cell and PNI had the highest AUC among lymphocyte subsets and nutritional markers, respectively. Therefore, we mainly investigated the predictive ability of CD19 (+) B cell binding PNI on the disease progression and clinical outcomes of gastric cancer patients. Correlation analysis found that CD19 (+) B cell–PNI was related to age, BMI, TNM staging, CA724, tumor size, and a wide range of blood parameters. Survival analysis showed that CD19 (+) B cell–PNI was not only associated with the prognosis of gastric cancer patients who underwent surgery, but also an independent prognostic factor for them. The nomograms containing CD19 (+) B cell–PNI also showed a high consistency between the predicted survival probability and the actual survival probability. These results all confirm its predictive value in gastric cancer. In addition, due to the cautious attitudes of doctors and patients towards endoscopic submucosal dissection (ESD), as well as some early metastases that cannot be detected by imaging examinations, our study included patients with all TNM stages. Although the significant correlation between CD19 (+) B cell combined with PNI and TNM stage resulted in uneven distribution of patients in different groups, we could still find that it had prognostic value in different TNM stages.

Some possible mechanisms could explain how CD19 (+) B cell combined with PNI could accurately predict the prognosis of gastric cancer patients. CD19 was a molecule that was expressed on all B cell lineages except for plasma cells. Belonging to the immunoglobulin superfamily, it played a critical role in B cell development, activation, and proliferation [36,37]. An increase in CD19 (+) B cells reflected an enhancement of humoral immunity in patients and was important in anti-tumor immunity [38,39]. On the one hand, tumor tissues could produce tumor-associated antigens, and antibodies produced by B cells bind to these antigens to induce antibody-dependent cell-mediated cytotoxicity. On the other hand, B cells could bind to tumor-associated antigens, process and present the antigen to induce T cell immune response, or interact with macrophages and complement systems to eliminate tumor cells [40,41]. Albumin not only reflected the nutritional status of patients but also indicated liver function reserve and treatment tolerance [42,43]. Additionally, the decrease in serum albumin was related to systemic inflammatory status, as cytokines produced during inflammation could both inhibit liver synthesis of albumin and induce albumin denaturation, leading to a rapid decrease in serum albumin levels [19,44,45]. Prolonged inflammation could also inhibit the function of the immune system, leading to tumor progression. Lymphocytes were the main participants in immune response, and the decrease in lymphocyte levels resulted in a reduction in anti-tumor immune response, leading to a more rapid development of tumors [46]. Therefore, albumin combined with lymphocytes could predict the prognosis of tumor patients.

However, single indicators had certain limitations in predicting patient prognosis. Although ALB can accurately reflect the patient’s status, it is influenced by various factors, including liver and kidney function, nutritional status, and inflammatory response. Changes in these factors can affect the level of ALB [47]. Additionally, patients with gastric cancer typically experience digestive symptoms, nausea, vomiting, and other issues, which may also impact their dietary intake and ALB levels [48]. Similarly, lymphocytes are influenced by factors such as infection, medication, nutritional status, and immune system diseases, leading to certain limitations [5]. CD19 (+) B cells reflect the immune function of a patient, but the immune function of cancer patients is also affected by various factors, such as inflammation and nutritional status, tumor activity, age, and psychological issues [49,50,51]. By combining PNI and CD19 (+) B cell measurements to predict patient prognosis, the limitations of using single indicators could be minimized, resulting in more accurate results. Overall, the combination of CD19 (+) B cells and PNI comprehensively assessed the patient’s status from the perspective of immunity, nutrition, and inflammation, and could accurately predict the clinical outcome of gastric cancer patients.

In this study, we were unable to eliminate the potential bias in information brought about by a single-center retrospective study. In addition, this study only focused on gastric cancer patients who underwent surgery, and the application of CD19 (+) B cells combined with PNI in other types of cancer requires further exploration in subsequent studies. Another issue worth noting is that the differences of CD19 (+) B cells and PNI among different types of cancer patients made them still lack a recognized cut-off value. Finally, the conclusions of this study need to be further verified by a larger sample size prospective experiment.

## 5. Conclusions

The lymphocyte subsets including CD3 (+) T cell, CD3 (+) CD8 (+) T cell, CD3 (+) CD16 (+) CD56 (+) NK T cell, and CD19 (+) B cell were related to the clinical outcomes of patients with gastric cancer who underwent surgery. Additionally, PNI combined with CD19 (+) B cell as a new biomarker had higher prognostic value than single markers and other non-invasive biomarkers. This combination could be used to identify patients with a high risk of metastasis and recurrence after surgery.

## Figures and Tables

**Table 1:** Patient characteristics.

	CD19 (+) B Cell–PNI Group			
	Group 1	Group 2	Group 3	*p* Value
Item	*n* = 56	*n* = 190	*n* = 45	
Age (years), mean (SD)	63.73 (10.56)	58.78 (9.80)	54.40 (10.85)	<0.001
Sex (%)				0.453
Male	41 (73.2)	134 (70.5)	28 (62.2)	
Female	15 (26.8)	56 (29.5)	17 (37.8)	
BMI (Kg/m^2^), mean (SD)	21.68 (3.29)	22.91 (3.00)	24.13 (3.56)	0.001
Radical resection (%)				0.191
Yes	52 (92.9)	177 (93.2)	45 (100)	
No	4 (7.1)	13 (6.8)	0 (0.0)	
Primary tumor site (%)				0.610
Upper 1/3	1 (1.8)	8 (4.2)	2 (4.4)	
Middle 1/3	4 (7.1)	26 (13.7)	8 (17.8)	
Low 1/3	42 (75.0)	135 (71.1)	31 (68.9)	
Whole	9 (16.1)	21 (11.1)	4 (8.9)	
Borrmann type (%)				0.193
I	2 (3.6)	21 (11.1)	9 (20.0)	
II	15 (26.8)	59 (31.1)	13 (28.9)	
III	36 (64.3)	97 (51.1)	20 (44.4)	
IV	3 (5.4)	13 (6.8)	3 (6.7)	
LNP (%)				0.080
Yes	34 (60.7)	83 (43.7)	21 (46.7)	
No	22 (39.3)	107 (56.3)	24 (53.3)	
Tumor size (%)				<0.001
<20 mm	0 (0.0)	14 (7.4)	15 (33.3)	
20–50 mm	21 (37.5)	90 (47.4)	13 (28.9)	
>50 mm	35 (62.5)	86 (45.3)	17 (37.8)	
Differentiation (%)				0.116
Poor	21 (37.5)	64 (33.7)	16 (35.6)	
Moderately	31 (55.4)	100 (52.6)	18 (40.0)	
Well	2 (3.6)	15 (7.9)	9 (20.0)	
Unknown	2 (3.6)	11 (5.8)	2 (4.4)	
Lauren type (%)				0.989
Intestinal	27 (48.3)	93 (48.9)	23 (51.1)	
Diffuse	10 (17.9)	35 (18.4)	6 (13.3)	
Mixed	17 (30.4)	53 (27.9)	14 (31.1)	
Unknown	2 (3.6)	9 (4.7)	2 (4.4)	
TNM stage (%)				0.032
I	13 (23.2)	83 (43.7)	21 (46.7)	
II	13 (23.2)	48 (25.3)	10 (22.2)	
III	24 (42.9)	53 (27.9)	12 (26.7)	
IV	6 (10.7)	6 (3.2)	2 (4.4)	
CEA (%)				0.310
<1.97 ng/mL	32 (57.1)	88 (46.3)	24 (53.3)	
≥1.97 ng/mL	24 (42.9)	102 (53.7)	21 (46.7)	
CA199 (%)				0.141
<10.19 U/L	24 (42.9)	93 (48.9)	28 (62.2)	
≥10.19 U/L	32 (57.1)	97 (51.1)	17 (37.8)	
CA724 (%)				0.001
<2.17 U/L	17 (30.4)	98 (51.6)	30 (66.7)	
≥2.17 U/L	39 (69.6)	92 (48.4)	15 (33.3)	
CA125II (%)				0.897
<10.21 U/L	28 (50.0)	96 (50.5)	21 (46.7)	
≥10.21 U/L	28 (50.0)	94 (49.5)	24 (53.3)	

BMI: body mass index; LNP: lymph node positive; CEA: carcinoembryonic antigen; CA199: carbohydrate antigen 199; CA724: carbohydrate antigen 724; CA125II: carbohydrate antigen 125II; PNI: Prognostic Nutritional Index.

**Table 2:** Blood parameters.

	CD19 (+) B Cell–PNI Group			
	Group 1	Group 2	Group 3	*p* Value
Item, Mean (SD)	*n* = 56	*n* = 190	*n* = 45	
ALT (U/L)	19.32 (10.63)	21.27 (13.34)	23.52 (14.57)	0.277
AST (U/L)	21.98 (10.25)	21.81 (7.97)	22.38 (7.14)	0.918
γ-GGT (U/L)	15.65 (8.55)	26.14 (21.07)	22.96 (20.77)	0.002
TBIL (μmol/L)	10.78 (6.07)	13.46 (8.86)	11.21 (6.04)	0.042
DBIL (μmol/L)	4.11 (2.25)	4.31 (1.61)	3.99 (1.76)	0.503
IDBIL (μmol/L)	6.68 (4.25)	8.40 (3.46)	7.26 (4.58)	0.006
TP (g/L)	59.89 (6.29)	69.35 (5.42)	69.49 (4.57)	<0.001
ALB (g/L)	35.13 (3.53)	41.87 (3.46)	42.15 (3.17)	<0.001
GLOB (g/L)	25.16 (3.71)	27.42 (3.94)	27.34 (2.84)	<0.001
PALB (mg/L)	218.44 (72.14)	283.77 (72.60)	280.02 (73.89)	<0.001
Urea (mmol/L)	5.83 (1.51)	6.21 (5.02)	6.22 (1.84)	0.823
CREA (μmol/L)	80.25 (15.54)	87.52 (44.46)	78.38 (17.46)	0.206
UA (μmol/L)	265.27 (95.65)	304.58 (86.40)	311.42 (81.33)	0.007
Glu (mmol/L)	5.17 (1.04)	5.30 (1.22)	5.20 (1.00)	0.745
WBC (10^9^/L)	6.34 (2.91)	6.79 (2.09)	7.04 (1.76)	0.257
NEU (10^9^/L)	4.46 (2.95)	3.99 (1.92)	4.12 (1.55)	0.357
Lym (10^9^/L)	1.29 (0.40)	2.11 (0.71)	2.21 (0.70)	<0.001
CD3 (+) (%)	82.63 (84.64)	68.60 (10.61)	66.08 (7.60)	0.036
CD3 (+) CD4 (+) (%)	41.89 (8.80)	40.41 (8.66)	41.19 (8.61)	0.507
CD3 (+) CD8 (+) (%)	24.26 (9.70)	23.86 (7.76)	20.50 (6.55)	0.029
CD4 (+)/CD8 (+)	2.10 (1.09)	1.96 (1.07)	2.34 (1.09)	0.094
CD3 (+) CD4 (+) CD8 (+) (%)	0.32 (0.36)	0.60 (1.47)	0.54 (0.69)	0.328
CD19 (+) (%)	9.58 (3.31)	10.01 (3.46)	19.02 (3.05)	<0.001
CD3 (−) CD16 (+) CD56 (+) (%)	15.70 (9.76)	18.27 (9.81)	11.45 (5.30)	<0.001
CD3 (+) CD16 (+) CD56 (+) (%)	3.51 (3.44)	3.04 (3.64)	3.02 (7.22)	0.761

ALT: alanine transaminase; AST: aspartate aminotransferase; γ-GGT: γ-glutamyl transferase; TBIL: total bilirubin; DBIL: direct bilirubin; IDBIL: indirect bilirubin; TP: total protein; ALB: albumin; GLOB: globulin; PALB: prealbumin; WBC: white blood cell; NEU: neutrophil; Lym: lymphocyte; PNI: Prognostic Nutritional Index.

**Table 3:** Univariate and multivariate analysis for PFS.

		PFS		
	Univariate Analysis		Multivariate Analysis	
Parameters	HR (95% CI)	*p*	HR (95% CI)	*p*
Age (years)	1.036 (1.015–1.057)	0.001	1.021 (1.000–1.042)	0.047
Sex				
Male	1 (Ref)			
Female	0.918 (0.601–1.401)	0.692		
BMI (Kg/m^2^)	0.931 (0.877–0.988)	0.018		
CD3 (+) (%)	1.003 (1.000–1.005)	0.051		
CD3 (+) CD4 (+) (%)	0.998 (0.975–1.021)	0.854		
CD3 (+) CD8 (+) (%)	1.027 (1.003–1.051)	0.026		
CD4 (+)/CD8 (+)	0.934 (0.769–1.134)	0.493		
CD3 (+) CD4 (+) CD8 (+) (%)	0.909 (0.715–1.155)	0.435		
CD19 (+) (%)	0.934 (0.893–0.978)	0.003		
CD3 (−) CD16 (+) CD56 (+) (%)	0.994 (0.973–1.015)	0.581		
CD3 (+) CD16 (+) CD56 (+) (%)	1.031 (1.002–1.061)	0.039		
ALB (g/L)	0.949 (0.909–0.990)	0.016		
Lym (10^9^/L)	0.690 (0.517–0.920)	0.012		
PNI	0.952 (0.924–0.982)	0.002		
CD19 (+) B cell–PNI				
Group 1	1 (Ref)		1 (Ref)	
Group 2	0.443 (0.293–0.670)	<0.001	0.763 (0.483–1.206)	0.248
Group 3	0.198 (0.088–0.447)	<0.001	0.352 (0.149–0.831)	0.017
Radical resection (%)				
Yes	1 (Ref)		1 (Ref)	
No	4.182 (2.335–7.492)	<0.001	1.411 (0.579–3.439)	0.448
Primary tumor site (%)				
Upper 1/3	1 (Ref)			
Middle 1/3	0.627 (0.196–2.000)	0.430		
Low 1/3	0.877 (0.320–2.401)	0.798		
Whole	2.084 (0.712–6.104)	0.180		
Borrmann type (%)				
I	1 (Ref)		1 (Ref)	
II	6.081 (1.448–25.533)	0.014	1.978 (0.400–9.783)	0.403
III	8.088 (1.977–33.091)	0.004	2.282 (0.476–10.928)	0.302
IV	28.997 (6.605–127.294)	<0.001	4.626 (0.877–24.383)	0.071
LNP (%)				
No	1 (Ref)		1 (Ref)	
Yes	3.537 (2.324–5.384)	<0.001	1.050 (0.511–2.158)	0.895
Tumor size (%)				
<20 mm	1 (Ref)		1 (Ref)	
20–50 mm	2.715 (0.831–8.870)	0.098	1.536 (0.402–5.871)	0.531
>50 mm	6.883 (2.165–21.878)	0.001	1.203 (0.576–1.431)	0.677
TNM stage (%)				
I	1 (Ref)		1 (Ref)	
II	3.875 (1.878–7.996)	<0.001	3.192 (1.388–7.340)	0.006
III	11.807 (6.187–22.533)	<0.001	8.472 (3.134–22.904)	<0.001
IV	45.844 (20.022–104.969)	<0.001	21.182 (6.246–71.836)	<0.001

HR: hazard ratio; BMI: body mass index; LNP: lymph node positive; PNI: Prognostic Nutritional Index; ALB: albumin; Lym: lymphocyte.

**Table 4:** Univariate and multivariate analysis for OS.

		OS		
	Univariate Analysis		Multivariate Analysis	
Items	HR (95% CI)	*p*	HR (95% CI)	*p*
Age (years)	1.037 (1.016–1.058)	<0.001	1.022 (1.001–1.043)	0.045
Sex				
Male	1 (Ref)			
Female	0.912 (0.597–1.392)	0.669		
BMI (Kg/m^2^)	0.932 (0.879–0.989)	0.021		
CD3 (+) (%)	1.003 (1.000–1.003)	0.028		
CD3 (+) CD4 (+) (%)	0.998 (0.976–1.021)	0.885		
CD3 (+) CD8 (+) (%)	1.028 (1.004–1.052)	0.023		
CD4 (+)/CD8 (+)	0.935 (0.771–1.135)	0.496		
CD3 (+) CD4 (+) CD8 (+) (%)	0.913 (0.720–1.158)	0.454		
CD19 (+) (%)	0.933 (0.892–0.977)	0.003		
CD3 (−) CD16 (+) CD56 (+) (%)	0.994 (0.937–1.015)	0.549		
CD3 (+) CD16 (+) CD56 (+) (%)	1.032 (1.002–1.063)	0.035		
ALB (g/L)	0.947 (0.908–0.989)	0.013		
Lym (10^9^/L)	0.684 (0.513–0.911)	0.009		
PNI	0.951 (0.922–0.980)	0.001		
CD19 (+) B cell–PNI				
Group 1	1 (Ref)		1 (Ref)	
Group 2	0.434 (0.287–0.656)	<0.001	0.721 (0.455–1.143)	0.164
Group 3	0.191 (0.085–0.430)	<0.001	0.319 (0.134–0.757)	0.010
Radical resection (%)				
Yes	1 (Ref)		1 (Ref)	
No	4.356 (2.431–7.807)	<0.001	1.769 (0.762–4.105)	0.184
Primary tumor site (%)				
Upper 1/3	1 (Ref)			
Middle 1/3	0.609 (0.191–1.944)	0.402		
Low 1/3	0.885 (0.323–2.423)	0.812		
Whole	2.056 (0.702–6.023)	0.189		
Borrmann type (%)				
I	1 (Ref)		1 (Ref)	
II	6.025 (1.435–25.300)	0.014	2.002 (0.405–9.909)	0.395
III	8.012 (1.958–32.780)	0.004	2.180 (0.454–10.467)	0.330
IV	27.087 (6.171–118.891)	<0.001	4.625 (0.876–24.410)	0.071
LNP (%)				
No	1 (Ref)		1 (Ref)	
Yes	3.445 (2.264–5.242)	<0.001	1.089 (0.532–2.232)	0.815
Tumor size (%)				
<20 mm	1 (Ref)		1 (Ref)	
20–50 mm	2.710 (0.829–8.858)	0.099	1.466 (0.386–5.568)	0.574
>50 mm	6.917 (2.176–21.988)	0.001	1.217 (0.546–1.355)	0.516
TNM stage (%)				
I	1 (Ref)		1 (Ref)	
II	3.833 (1.858–7.910)	<0.001	3.282 (1.435–7.505)	0.005
III	11.441 (5.999–21.819)	<0.001	9.280 (3.441–25.029)	<0.001
IV	35.899 (15.895–81.079)	<0.001	15.617 (4.770–51.134)	<0.001

HR: hazard ratio; BMI: body mass index; LNP: lymph node positive; PNI: Prognostic Nutritional Index; ALB: albumin; Lym: lymphocyte.

**Table 5:** The calculation formulas.

Items	Calculation Formulas
GNRI	[1.519 × albumin (g/L)] + [41.7 × (weight/Wlo)]
NRI	[1.489 × albumin (g/L)] + [41.7 × (weight/Wlo)]
SII	platelet (10^9^/L) × neutrophil (10^9^/L)/lymphocyte (10^9^/L)
SIRI	Monocyte (10^9^/L) × neutrophil (10^9^/L)/lymphocyte (10^9^/L)
ALI	BMI (Kg/m^2^) × albumin (g/dL) × lymphocyte (10^9^/L)/neutrophil (10^9^/L)

GNRI, geriatric nutritional risk index; NRI, nutritional risk index; SII, systemic immune-inflammation index; SIRI, systemic inflammation response index; ALI, advanced lung cancer inflammation index; The Lorentz equations (Wlo) were as follows: male = Height − 100 − [(Height − 150)/4]; female = Height − 100 − [(Height − 150)/2.5].

**Table 6:** The AUC of different parameters.

Parameters	AUC	95% CI
CD19 (+) B cell–PNI	0.648	0.582–0.713
Age	0.621	0.555–0.687
BMI	0.584	0.517–0.651
Differentiation	0.562	0.494–0.629
TNM stage	0.817	0.766–0.868
Lauren type	0.537	0.469–0.605
Tumor size	0.668	0.605–0.731
Primary tumor site	0.571	0.502–0.640
Borrmann type	0.646	0.582–0.711
NRI	0.593	0.525–0.661
GNRI	0.591	0.523–0.598
PNI	0.615	0.547–0.683
SII	0.567	0.498–0.637
SIRI	0.561	0.491–0.631
ALI	0.536	0.466–0.607
ALT	0.533	0.465–0.602
AST	0.504	0.436–0.572
γ-GGT	0.533	0.465–0.601
TBIL	0.582	0.513–0.651
DBIL	0.547	0.478–0.615
IDBIL	0.586	0.518–0.655
TP	0.583	0.515–0.652
ALB	0.580	0.511–0.648
GLOB	0.542	0.473–0.611
A/G	0.533	0.464–0.601
PALB	0.640	0.599–0.727
Urea	0.516	0.446–0.586
CREA	0.537	0.467–0.607
UA	0.549	0.478–0.621
Glu	0.538	0.469–0.607
WBC	0.526	0.456–0.597
NEU	0.514	0.443–0.585
Lym	0.606	0.538–0.675
CEA	0.563	0.494–0.631
CA199	0.543	0.474–0.612
CA724	0.610	0.543–0.677
CA125II	0.588	0.520–0.655
CD3 (+)	0.582	0.511–0.652
CD3 (+) CD4 (+)	0.500	0.429–0.571
CD3 (+) CD8 (+)	0.564	0.494–0.632
CD4 (+)/CD8 (+)	0.533	0.462–0.603
CD3 (+) CD4 (+) CD8 (+)	0.511	0.442–0.579
CD19 (+)	0.601	0.534–0.668
CD3 (−) CD16 (+) CD56 (+)	0.536	0.466–0.607
CD3 (+) CD16 (+) CD56 (+)	0.546	0.475–0.617

AUC: area under curve; CI: confidence interval; PNI: Prognostic Nutritional Index; GNRI: geriatric nutritional risk index; NRI: nutritional risk index; SII: systemic immune-inflammation index; SIRI: systemic inflammation response index; ALI: advanced lung cancer inflammation index; ALT: alanine transaminase; AST: aspartate aminotransferase; γ-GGT: γ-glutamyl transferase; TBIL: total bilirubin; DBIL: direct bilirubin; IDBIL: indirect bilirubin; TP: total protein; ALB: albumin; GLOB: globulin; PALB: prealbumin; Urea: urea nitrogen; CREA: creatinine; UA: uric acid; Glu: glucose; WBC: white blood cell; NEU: neutrophil; Lym: lymphocyte; CEA: carcinoembryonic antigen; CA199: carbohydrate antigen 199; CA724: carbohydrate antigen 724; CA125II: carbohydrate antigen 125II.

## Data Availability

The authors promise to provide the original data supporting this study without reservation.
